# Wine Storage at Cellar vs. Room Conditions: Changes in the Aroma Composition of Riesling Wine

**DOI:** 10.3390/molecules26206256

**Published:** 2021-10-16

**Authors:** Andrii Tarasov, Federico Garzelli, Christoph Schuessler, Stefanie Fritsch, Christophe Loisel, Alexandre Pons, Claus-Dieter Patz, Doris Rauhut, Rainer Jung

**Affiliations:** 1 Department of Enology, Hochschule Geisenheim University, Von-Lade-Str 1, 65366 Geisenheim, Germany; federico.garzelli@edu.unito.it (F.G.); Christoph.Schuessler@hs-gm.de (C.S.); Rainer.Jung@hs-gm.de (R.J.); 2 Department of Microbiology and Biochemistry, Hochschule Geisenheim University, Von-Lade-Str 1, 65366 Geisenheim, Germany; Stefanie.Fritsch@hs-gm.de (S.F.); Doris.Rauhut@hs-gm.de (D.R.); 3 R&D and Oenology Center, DIAM Bouchages SAS, Espace Tech Ulrich, 66400 Ceret, France; loisel@diam-bouchage.com; 4 Unité de Recherche Œnologie, EA 4577, USC 1366 INRAE, Université de Bordeaux, Bordeaux INP, 33882 Villenave d’Ornon, France; alexandre.pons@u-bordeaux.fr; 5 Seguin Moreau Cooperage, ZI Merpins, 16100 Cognac, France; 6 Department of Beverage Research, Hochschule Geisenheim University, Von-Lade-Str 1, 65366 Geisenheim, Germany; Claus.Patz@hs-gm.de

**Keywords:** Riesling, wine, temperature, sulfur dioxide, oxygen, storage, aging, stopper, aroma, sensory

## Abstract

Storage temperature is one of the most important factors affecting wine aging. Along with bottling parameters (type of stopper, SO_2_ level and dissolved O_2_ in wine), they determine how fast wine will evolve, reach its optimum and decline in sensory quality. At the same time, lowering of the SO_2_ level in wine has been a hot topic in recent years. In the current work, we investigated how Riesling wine evolved on the molecular level in warm (~25 °C) and cool (~15 °C) conditions depending on the SO_2_ level in the wine (*low*, *medium* and *high*), flushing of the bottle’s headspace with CO_2_ and three types of stoppers (*Diam 30*, *Diam 30 origin* and *Diam 5*) with different OIR levels (0.8–1.3 mg) and OTR levels (0.3–0.4 mg/year). It was demonstrated that the evolution of primary and secondary aromas, wine color and low molecular weight sulfur compounds (LMWSCs) during the two years of aging mainly depended on the storage temperature. Variation in the SO_2_ level and CO_2_ in the headspace affected mostly certain LMWSCs (H_2_S, MeSH) and β-damascenone. New aspects of C_13_-norisprenoids and monoterpenoids behavior in Riesling wine with different levels of SO_2_ and O_2_ were discussed. All three types of stoppers showed very close wine preservation properties during the two years of storage. The sensory analysis revealed that, after only six months, the warm stored wines with a *low SO_2_* level were more oxidized and different from the samples with *medium* and *high* SO_2_ levels. A similar tendency was also observed for the cool stored samples.

## 1. Introduction

Wine plays an important role in many cultures, being not just a drink but also an instrument of social relations and aesthetic experience [1]. There are various scenarios in which people buy and consume wine, including giving it as a present, or keeping it for special occasions, etc. Therefore, the question of wine storage is pertinent for not only wine producers and retailers, but also for consumers. For their part, consumers usually have limited possibilities for storing wines and often hold them under room conditions at temperatures of above 20 °C. Higher temperatures are known to accelerate chemical reactions and lead to faster wine development in the bottle [2,3]. Thus, the goal of our current research was to investigate transformations in the wine aroma composition at warm vs. cool storage conditions, and across several different wine bottling strategies. The bottling variants differed in terms of the SO_2_ level in wine, CO_2_ treatment of the bottle’s headspace and application of three types of stoppers. Riesling wine was chosen for this experiment due its special chemical composition (discussed below) and its importance for both local and global wine industries.

Studies related to the bottle aging of white wines are usually focused on their aroma substances and wine color changes [4,5]. Many white wines are recommended to be consumed young—ideally within one year of bottling. This recommendation is related to their aroma composition, which is represented mainly by secondary (fermentation) aromas and poor in primary (varietal) aromas. The most prominent compounds among the secondary wine aromas are esters, which impart an intense fruity character to young wines [6,7]. However, esters (especially acetate esters) are unstable at wine pH and hydrolyze relatively fast, yielding much less odor-potent products, acids, and alcohols, which results in a loss of fruity aroma. For example, the content of isoamyl acetate (ice candy, pineapple aroma) can drop more than two times after only half a year of storage. In general, a lower pH and higher temperature accelerate the hydrolysis processes [8,9,10].

There is also another group of white wines, which can be suitable for longer storage. These wines are rich in varietal aromas and are made from grape varieties, such as Riesling, Gewürztraminer, Sauvignon Blanc, Muscat, etc. [11]. The content of many primary varietal compounds in wines is usually more stable during the bottle aging, compared to the secondary aroma esters. Moreover, the products of chemical transformations of primary aromas can also be odorous, which enrich the wine bouquet with tertiary (aging-related) aromas [12]. All of these phenomena make wines rich in varietal aromas more suitable for longer storage. Terpenes, C_13_-norisiprenoids, pyrazines and (polyfunctional) thiols are the main families of chemical substances responsible for the varietal aromas.

In general, there are two main drivers of the chemical transformation of wine components:

- *Oxygen dissolved in wine*, which causes the oxidation reactions of aroma compounds, phenolics and other wine substances [13]. It was demonstrated that oxidation reactions in wine are catalyzed by copper and iron ions [14]. These metals induce the formation of a hydroperoxyl radical, which launches the cascade of oxidation processes via formation of quinones, hydrogen peroxide and following intermediates. These processes describe non-enzymatic oxidation, which occurs in the bottled wine. The addition of SO_2_ to a wine aims to chemically reduce H_2_O_2_ and quinones in order to protect wine aromas and phenolic compounds [13].
- *Low wine pH*, which firstly facilitates non-oxidative intra-molecular rearrangements, that are typical for certain primary aroma compounds, especially terpenes and C_13_-norisoprenoids. However, their mechanisms are not yet completely studied. Second, *low wine pH* induces hydrolysis processes in wine, which are relevant for secondary aroma esters and some primary aroma compounds with ester fragments, e.g., polyfunctional thiol 3-sulfanylhexyl acetate (3-SHA) [9,13]. In addition, non-enzymatic hydrolysis of certain precursors leads to a release of varietal aroma compounds during bottle aging [15].

In view of these chemical transformations, pyrazines seem to be the most stable compounds among the primary wine aromas. It has been shown that pyrazines content slowly decreases during wine storage and that this is mainly due to the scalping effects of bottle closures [16]. At the same time, light exposure of the wines and temperature conditions did not affect the pyrazines concentration significantly [17]. In its turn, polyfunctional thiols demonstrate the opposite properties, as they are sensitive to oxidation processes in wine. As a result, non-odorous compounds are formed (e.g., reaction products with quinones), which leads to a lower wine aroma intensity. Wines from grape varieties rich in thiols, such as Sauvignon Blanc, also possess a considerable amount of precursors of polyfunctional thiols. However, it does not seem that these precursors can be hydrolyzed non-enzymatically during bottle aging, yielding noticeable amounts of free thiols in addition [13,18].

In contrast to pyrazines and thiols, much more diverse chemical reactions were observed for monoterpenoids and C_13_-norisoprenoids. First, noticeable amounts of these compounds can be additionally released due to the hydrolysis of precursors during bottle aging. This allows even for increasing the concentrations of free monoterpenoids and C_13_-norisoprenoids in some wines during the initial steps of bottle aging. Second, these compounds undergo numerous chemical transformations in wine, producing new substances, including tertiary (aging-related) aromas. For example, it was demonstrated, that nerol, geraniol, linalool, and β-citronellol usually contribute to the monoterpenoids composition of fruity young wines, while 1,8-cineole, 1,4-cineole, α-terpineol, terpinen-4-ol are more typical for aged wines with a developed bouquet [13,17,19]. As for C_13_-norisoprenoids, β-damascenone and β-ionone usually provide fruity and floral aromas to young wines and are replaced during aging by other aromas, such as kerosene and camphor, due to the formation of 1,1,6-trimethyl-1,2-dihydronaphthalene (TDN) and vitispirane, respectively [17]. The latter compounds are especially typical for aged Riesling wines, which have been extensively studied in recent decades.

The above-mentioned facts make monoterpenoids and C_13_-norisoprenoids interesting molecules for the study of wine aging processes. This topic is also pressing, because there is still a lot of missing information about the mechanisms of monoterpenoids and C_13_-norisoprenoids aging transformations, including the role and importance of oxygen in these processes. One of the few wines which possess important amounts of both, monoterpenoids and C_13_-norisoprenoids, is Riesling. Therefore, Riesling wine was especially suitable for studying the aging transformations of wine aroma.

In order to summarize the aim of our work, the following questions were defined for investigation:

1. What is the impact of the SO_2_ level on wine development, including wine aroma?
2. How efficiently can wine freshness be preserved in the variants with a *low SO_2_* level (sensory evaluation)?
3. What is the role of oxygen and SO_2_ in the chemical transformations of wine aromas, specifically monoterpenes/monoterpenoids and C_13_-norisoprenoids?
4. How do bottling and storage conditions (cool and warm) affect varietal and secondary aromas in wine, as well as low molecular weight sulfur compounds (LMWSCs)?
5. Does the type of studied bottle stoppers affect the wine parameters after two years of aging?

## 2. Materials and Methods

### 2.1. Wine Bottling and Experiment Design

**Figure 1:**
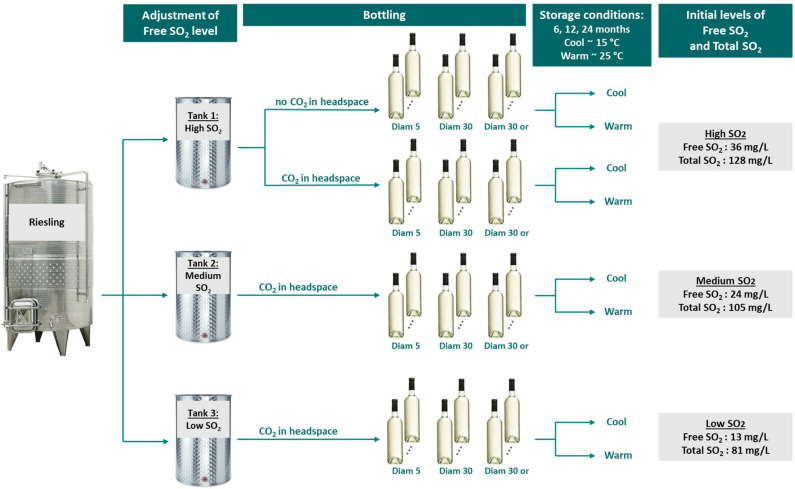
Design of the experiment with Riesling wine: bottling and storage scheme.

Typical Riesling white wine from the Rheingau region (Germany) was used for the experiment. The wine was produced and bottled for this study in the summer of 2018 in the facilities of the Hochschule Geisenheim University (Germany) winery. Prior to bottling, the wine was transferred into three smaller tanks to adjust different levels of Free SO_2_ (with 5% SO_2_ water solution) as *high*, *medium,* and *low*. Transparent glass bottles of 0.75 L (produced by “Verallia” and supplied by Saint Gobain-Oberland, Germany) were filled up to 63 mm ± 5 mm from the top of the bottleneck and the headspace of part of the bottles was flushed with CO_2_ according to the experiment design (Figure 1). The corker machine GAI 4040 was used to insert three types of micro-agglomerated stoppers (38 mm length): *Diam 5*, *Diam 30* and *Diam 30 origin*. No capsules were applied above the stoppers.

*Diam 5* and *Diam 30* are composed of a blend of cork granules with a size of 0.35–1.5 mm, food grade polyurethane binder and food grade Expancel microspheres. The main difference between *Diam 5* and *Diam 30* is the cork granules/binder ratio, which affects the stopper’s density (280 ± 20 kg/m^3^ and 320 ± 20 kg/m^3^, respectively) and oxygen permeability. The latter parameter defines the recommended storage time for wine: five years for *Diam 5* and thirty years for *Diam 30* stoppers. The oxygen initial rate (OIR) is 0.8 mg for *Diam 30*, 1.1 mg for *Diam 30 origin* and 1.3 mg for *Diam 5*. The oxygen transmission rate (OTR) is 0.4 mg/year for *Diam 5* closures and 0.3 mg/year for *Diam 30* and *Diam 30 origin* stoppers. The particularities of the *Diam 30 origin* closures are bio-based binder (aliphatic isocyanates + polyols from castor oil) and beeswax, instead of Expancel microspheres, which reduce the wine capillarity inside the stoppers. The *Diam 30 origin* density is the same as *Diam 30* (320 ± 20 kg/m^3^). All three types of *Diam* stoppers are coated with a blend of paraffin and silicone.

After the bottling process, the bottles were kept upright until the next day to allow the closures to adjust to the bottlenecks and prevent wine leakage. All of the bottles were then placed horizontally into cardboard cases. To avoid light exposure of the bottles, the cases were closed and sealed with an adhesive tape. The cases were placed on wooden pallets and were stored in cool (15 ± 3 °C, 55 ± 6% humidity) and warm (25 ± 3 °C, 29 ± 7% humidity) conditions. These conditions were chosen to provide a temperature difference of about 10 °C and simulate wine storage in a cellar and in a residential apartment room, respectively. The storage conditions were automatically monitored on a daily basis by means of the multifunctional logger, Bosch TDL 110.

As a result, 24 bottling/storage variants were produced, which differed in the level of SO_2_ in the wine, CO_2_ treatment of the headspace, storage temperature and three types of stoppers. The initial levels of SO_2_ (one week after bottling) are indicated in Figure 1. The wines were analyzed at three storage points: 6, 12 and 24 months of storage. It is worth noting that the current article is a part of the long-term ongoing project, which involves the study of the storage properties of three types of wine stoppers, *Diam 5*, *Diam 30*, and *Diam 30 origin*. These stoppers are designed for multi-year bottle aging, five and thirty years, respectively. Thus, after the analysis of wines stored for two years, the variants, which differed only in the stopper type, demonstrated very similar results for all physicochemical analyses 2.2–2.6. Therefore, to achieve a convenient and clearer presentation of the results, the wines, which differed only in the stopper type, were grouped, and are considered as replicates in the Section 3. In this way, the bottling storage variants were reduced from 24 to 8, and were as follows:

- *High SO_2_/no CO_2_/cool;*
- *High SO_2_/no CO_2_/warm;*
- *High SO_2_/CO_2_/cool;*
- *High SO_2_/CO_2_/warm;*
- *Medium SO_2_/CO_2_/cool;*
- *Medium SO_2_/CO_2_/warm;*
- *Low SO_2_/CO_2_/cool;*
- *Low SO_2_/CO_2_/warm.*

### 2.2. Analysis of Oxygen in the Bottles’ Headspace and Oxygen Dissolved in Wine

The concentration of dissolved oxygen in the wine (ppb) and the oxygen content in the bottle’s headspace (partial pressure, hPa) were measured by a non-destructive method using the fiber optical probe Fibox 3 LCD trace (PreSens ^®^ Precision Sensing GmbH, Regensburg, Germany) [20,21]. The oxygen-reactive polymeric sensor-spots, PSt3, were glued inside the bottles on the level of expected headspace (no contact with the wine after bottling) and in the lower part of the bottles (in contact with wine). In total, 12 additional bottles with two sensor-spots were bottled: six bottles *High SO_2_/CO_2_/cool* (with three types of stoppers in two replicates), and six bottles *High SO_2_/no CO_2_/cool* (with three types of stoppers in two replicates). These bottles were stored together with the main batch of the bottles in cool conditions. The measurements were taken for a total of six months: each second-third day during the first two months, and at least once a week during the last four months. The resulting values were calculated as the means of two replicates and are presented in the graphs in Section 3.1.

### 2.3. General Wine Analysis

The wines for the general wine analysis and all physicochemical analyses mentioned below were sampled immediately after opening the bottles, which were preliminary cooled by 4 °C. The wines were analyzed directly after sampling or were stored in the fridge (4 °C) in 100 mL brown glass bottles with screw caps (PTFE liners) before analysis.

#### 2.3.1. FTIR Analysis

Basic wine parameters, including Free and Total SO_2_ content, were measured using the Winescan™ SO_2_ Foss instrument and Foss Integrator software (Foss, Hillerød, Denmark) in accordance with the analytical method described in previous publications [22,23,24].

#### 2.3.2. Analysis of Acetaldehyde

Acetaldehyde concentrations in the wine samples were determined by the enzymatic method K-ACHYD (Megazyme, Chicago, IL, USA) according to the instructions [25], involving spectrophotometric analysis by means of Spectrophotometer evolution 220 (Thermofisher-Scientific, Waltham, MA, USA).

#### 2.3.3. Color Measurement

PhotoLab 7600 UV-VIS spectrophotometer (XylemAnalytics GmBH, Weilheim in Oberbayern, Germany) was utilized to measure the UV-Vis spectra of the wine samples against the blank (distilled water), using cuvettes with a path length of 10 mm. The spectra were acquired over a visible region of 380 to 770 nm and the CIELAB color space was evaluated.

### 2.4. Analysis of Monoterpenes, Monoterpenoids and C_13_-Norisoprenoids

The determination of free terpenes and C_13_-norisoprenoids was conducted by means of headspace solid phase microextraction (HS-SPME) in connection with gas chromatography and mass spectrometry (GC-MS), according to the method of Câmara et al. [26] and corresponding adaptations by Brandt et al. [27]. The following analytical equipment was applied: a multipurpose sampler MPS 2 (Gerstel GmbH & Co. KG, Mülheim an der Ruhr, Germany) was used for HS-SPME injection in combination with a GC-MS-System (GC 6890 and an MS 5973 (Agilent)). HS-SPME extraction was carried out with a 1 cm SPME fiber coated with 100 µm of polydimethylsiloxane (Supelco). Aroma compound separation was performed using a 30 m × 0.25 mm × 0.5 µm gas chromatography column (DB-Wax, J & W Scientific, Agilent) together with the mass spectrometer used in the SIM mode. For the instrumental control, acquisition and quantitative analysis of data, Agilent MassHunter workstation software was used. Examples of extracted ion chromatograms for TDN and linalool are provided in Supplementary Figure S1A,B.

### 2.5. Analysis of Secondary Wine Aromas

The main volatile substances related to the fermentation bouquet or secondary wine aroma compounds (esters, higher alcohols, fatty acids, etc.) were analyzed using headspace solid phase microextraction (HS-SPME), together with gas chromatography and mass spectrometry (GC-MS; (7890A and MS 5977B, Agilent)) using the multipurpose sampler MPS2 (Gerstel GmbH & Co. KG) for HS-SPME injection [23]. The determination followed the analytical method [28] according to the reported approach [26,29], which was correspondingly adjusted and adapted [30]. A model wine (10% (*v/v*) solution of ethanol in water, 3 g/L of tartaric acid, adjusted to pH 3) was used for the calibration. SPME extraction was performed with a 65 µm polydimethylsiloxane and divinylbenzene fiber (Supelco). The gas chromatography column of 60 m × 0.25 mm × 1 µm (Rxi-5Sil, Restek, Bellefonte, PA, USA), together with particular GC-MS software and technical settings, was utilized for the aroma compound separation. The Agilent MassHunter workstation software provided by the GC-MS instrumentation was used for the instrumental control, acquisition of data and analysis of qualitative and quantitative data.

### 2.6. Analysis of Low Molecular Weight Sulfur Compounds (LMWSCs)

The analysis required cooling of the wine bottles by 4 °C and analysis of the wine immediately after sampling directly from the bottle. The analytical approach involving headspace (HS) injection and the usage of gas chromatography (GC) and pulse flame photometric detection (PFPD) was used for the determination of ten LMWSCs: hydrogen sulfide (H_2_S), methanethiol (MeSH), ethanethiol (EtSH), dimethyl sulfide (DMS), carbon disulfide (CS_2_), thioacetic acid-S-methyl ester (MeSAc), thioacetic acid-S-ethyl ester (EtSAc), dimethyl disulfide (DMDS), diethyl disulfide (DEDS), and dimethyl trisulfide (DMTS). More details about the actual analysis are described in the recent publication [23].

### 2.7. Sensory Analysis

Two types of sensory analyses are presented in the current paper, which were performed at the same time points as physicochemical analyses. The goal was to evaluate the effects of SO_2_ level in wine and CO_2_ treatment of the bottlenecks on the wine development. The sensory tests were carried out separately for the cool and warm stored wines. The panels at each time point (6, 12 and 24 months) consisted of 14–17 participants, who were employees and students of the Hochschule Geisenheim University (Germany). No special training or panelist selection was done, since all of them had extensive wine experience, including in terms of Riesling wines. The sensory tests were performed in the specialized, well-lit, and odor-free sensory analysis room in the Department of Enology of Hochschule Geisenheim University, equipped with 30 individual booths. Paper questionnaires and wine tasting glasses (ISO 3591) with about 35 mL of the wine samples were located in front of each panelist in the booths. All of the wine bottles were labeled with a random three-digit number and were cooled in advance in order to reach a serving temperature of 12–13 °C.

**Figure 2:**
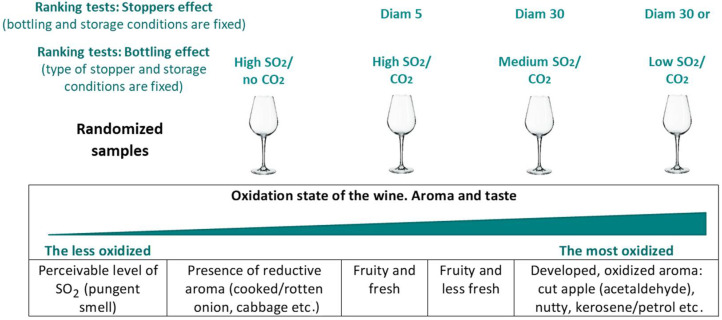
Design of the sensory analysis: *ranking tests*.

*Ranking tests* were carried out after 6, 12, and 24 months to compare the effect of the stoppers, and after six months of storage for comparison of the bottling effect. Each test comprised three or four glasses of the wine samples (Figure 2), which were randomized for each taster. The task for the panelists was to arrange the offered wines according to their oxidation state: the least oxidized wine—place 1, and the most oxidized sample—place 3 or 4 (depending on the number of glasses). Prior to the tasting, the concept of wine oxidation was discussed with the participants and the table from Figure 2 was given as a guidance.

**Figure 3:**
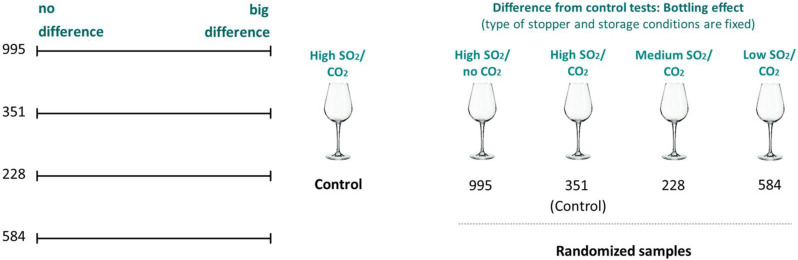
Design of the sensory analysis: *difference from control tests*.

*Ranking tests* are an instrument to detect a perceivable difference between the wines, however they do not show how big the difference is between the samples. In order to focus on the more precise differences after 12 and 24 months of storage, the *difference from control tests* [31] were applied. There was no stress on any specific wine aroma or taste descriptors in these tests, however the panelists were asked to pay attention to the oxidation state of the samples. The task was to evaluate how different the four presented wines were from the control and to indicate this as a dash on the continuous scale (Figure 3). *High SO_2_/CO_2_* variant was taken as the control, since this wine was expected to be the least oxidized in the test. The blind-control sample *high SO_2_/CO_2_* was present also among the randomized samples and it was expected that this sample would be indicated by the panelists close to the beginning of the scale.

### 2.8. Processing of the Data

Microsoft Office Standard 2013 programs (Version 15.0.5153.1000, Microsoft Corporation, Redmond, Washington, DC, USA) were used for the data treatment and preparation of figures. Fizz software 2.51 a 86 (2016, Biosystemes, Couternon, France) was utilized for the sensory analysis experiments: preparation and processing of questionnaires, and statistical analysis of the data. Friedman tests (sum of ranks) were applied to detect statistical differences in the *ranking tests*. In the case of the *difference from control tests*, the medians and means of the observed size of the difference from the control were calculated for each variant of wine and for the blind-control samples. The analysis of variance (ANOVA) procedure was applied in order to test the results for statistical significance. PCA analysis for the 24 months stored wine samples was undertaken using software R version 4.1.0 [32] with packages R Commander [33] and FactoMineR [34]. Statistical analysis in Section 3.2 was carried out using the software JASP (Version 0.14.1, [35]). Analysis of variance (ANOVA) was performed with the Tukey HSD test for post hoc comparison to discriminate among the means of Absorbance at 420 nm.

## 3. Results and Discussion

The presentation and discussion of the results is organized in accordance with the chronological actions and types of the analyzed wine aroma compounds.

### 3.1. Oxygen Content Inside the Bottles

**Figure 4:**
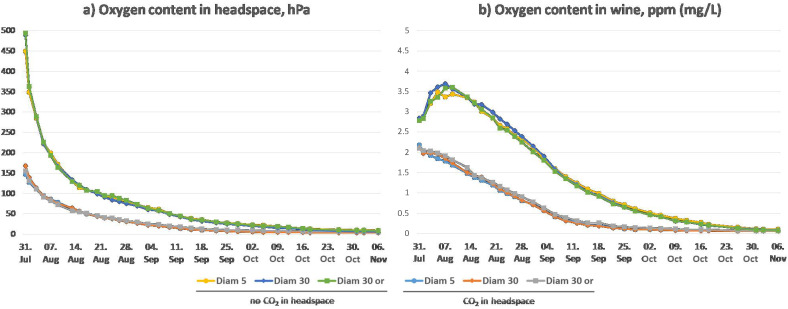
Measurement of the oxygen content inside the bottles: (**a**) oxygen content in the headspace, hPa; (**b**) oxygen content dissolved in wine, ppm (mg/L).

The flushing of the bottles’ headspace with CO_2_ demonstrated its efficiency. The measurements on the same day of bottling revealed that the oxygen content in the CO_2_–treated headspaces was about three times lower compared to that of the non-treated ones: about 150–170 hPa vs. 450–500 hPa, respectively (Figure 4). The concentration of dissolved O_2_ in wine also started to differ after bottling and a few hours later was 2.1–2.2 mg/L for the CO_2_–treated variants and about 2.8 mg/L for the non-treated bottles. The oxygen consumption by the wine led to the decrease in the content of dissolved O_2_ in the wine, except during the first days for those bottles without CO_2_ treatment. During this period, the process of O_2_ dissolution from the air was faster than the oxygen consumption by the wine, and after ten days the level of dissolved oxygen hit its maximum of 3.4–3.6 mg/L. Starting from this time point, all of the oxygen curves were declining and after about three months reached values close to zero. At that moment, most of the O_2_ present inside the bottle was consumed by wine, however this does not mean that the oxidation processes stopped. As was mentioned in the Introduction, O_2_ does not oxidize the wine components directly, but does so via intermediates, such as peroxide and other products, which do not disappear immediately. Moreover, the constant migration of oxygen through the stoppers also continues, but at a very low rate, typically measured in µg/L/day [13].

A relatively fast oxygen consumption, as in our case, is typical for many young wines, because they contain high concentrations of oxygen-reactive species that diminish the oxygen content quite quickly when bottled [13]. Another important aspect of this analysis is that the wines with all three types of Diam stoppers demonstrated very similar outcomes in terms of the oxygen consumption rate, which will be also discussed in the next Subsection.

### 3.2. General Wine Analysis

The main aim of the general wine analysis was to monitor the changes in the Free and Total SO_2_ levels in wines. In addition, the analyzing of other physicochemical parameters assisted in proving that no principal changes occurred in the wines during the aging process, e.g., due to unexpected microbiological activity or packaging defects. FTIR, color measurement and acetaldehyde analysis were used for this purpose.

**Figure 5:**
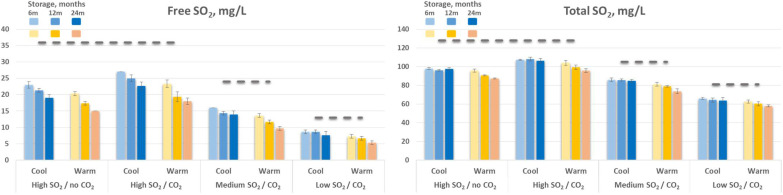
Concentration of Free and Total SO_2_ in the wines after 6, 12 and 24 months of storage (mean of three bottles with *Diam 5*, *Diam 30* and *Diam 30 origin* stoppers ± standard deviation). Dotted lines correspond to the initial levels of SO_2_ after bottling.

The loss of Free and Total SO_2_ clearly correlated with the storage temperature and CO_2_ in the headspace. The fastest decline of Free SO_2_ content was observed during the first six months (Figure 5), which is explained by the quick oxygen consumption by the wine (Figure 4). The most protected variant *High SO_2_/CO_2_* lost about 9 and 13 mg/L of Free SO_2_ at cool and warm storage conditions, respectively. No CO_2_ treatment of the bottle headspace resulted in an additional decrease by 3–4 mg/L of Free SO_2_ after six months (*High SO_2_/no CO_2_* variant). In the case of samples with *medium SO_2_* content, the fall of Free SO_2_ was about 8 mg/L for cool and 10 mg/L for warm storage.

Comparing the Free SO_2_ losses mentioned above with the Total SO_2_ ones, the latter were about twice as high (for *high* and *medium SO_2_* samples). This phenomenon is related to the partial replenishing of free SO_2_ due to the hydrolysis of bound SO_2_ forms. The decrease in Free SO_2_ for the samples with *high* and *medium SO_2_* at the next checkpoints after 12 and 24 months was about 2–4 mg/L. As was reported, the main source of new oxygen in the bottle during the first six months is the oxygen released from the compressed Diam stoppers in a total amount of about 0.8–1.3 mg (OIR) [36]. Later, the oxygen migration through the stoppers (OTR), at a rate about 0.3–0.4 mg/year, starts to play a key role. In turn, it was shown that the loss of SO_2_ in relation to O_2_ in oxidation reactions approaches the molar ratio of 2:1 or exceeds it depending on the wine type [37,38,39]. The SO_2_ losses in our wine samples were comparable to the reported ones. The possible reactions of SO_2_ with electrophiles other than H_2_O_2_ and quinones, as well as the warm storage (which leads to faster oxygen migration and oxidation processes) should also be taken into consideration.

In terms of Free SO_2_ levels and practical aspects, the *high* and *medium SO_2_* wines stored at cool conditions were still relatively well protected after two years, while the warm stored samples with *medium SO_2_* were already on the risky edge, having about 10 mg/L of Free SO_2_. As for the *low SO_2_* wines, the level of Free SO_2_ was already critical after six months, especially for the warm stored samples which reached below 8 mg/L of Free SO_2_. The *low SO_2_* samples in this experiment reflected the enological approach of wines with limited sulfur dioxide addition. According to the literature, low SO_2_ may lead to alterations in the wine aroma composition already after three months of storage [40]. In addition, it is noteworthy that the differences in SO_2_ levels between the wines bottled with *Diam 5*, *Diam 30* and *Diam 30 origin* stoppers were minimal. It is clear from the small standard deviations (Figure 5) that all of the tested stoppers protected the wine equally well after two years of storage.

Besides the SO_2_ content, no unusual changes in general were observed in the basic wine parameters during 24 months of storage (FTIR analysis). This can be assumed from the values and standard deviations in the Supplementary Table S1. The only wine component which varied was tartaric acid. Thus, about 0.5 g of tartaric acid precipitated in the cool stored samples, which also caused deviations in the other related wine parameters, such as Extract, Sugar-free Extract and Total acidity. Bottling parameters, such as SO_2_ level, CO_2_ in the headspace or type of the stopper did not influence the amount of precipitated tartaric acid.

Enzymatic analysis of acetaldehyde showed that its concentration was stable throughout the storage period. The values were about 24–25 mg/L regardless of the storage and bottling conditions (Supplementary Table S1). Despite none of the wine variants demonstrating elevated contents of acetaldehyde, this does not mean that no other wine components underwent oxidation, including aroma compounds and other substances (e.g., phenolic compounds). Moreover, the newly formed acetaldehyde is itself a reactive compound and can participate in further chemical transformations.

**Figure 6:**
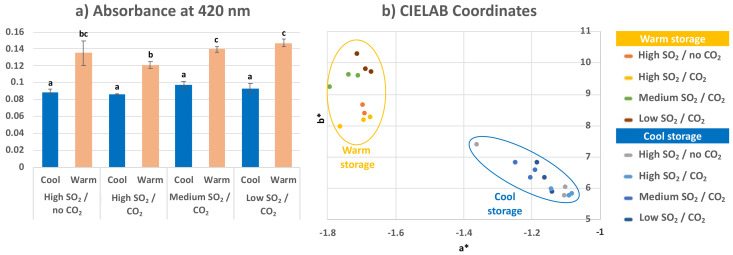
Color parameters of the wines after 24 months of storage: (**a**) Absorbance at 420 nm (mean of three samples with *Diam 5*, *Diam 30* and *Diam 30 origin* stoppers ± standard deviation); small letters denote significant differences according to the Tukey’s test (* *p* < 0.05); (**b**) CIELAB coordinates (three samples for each variant with *Diam 5*, *Diam 30* and *Diam 30 origin* stoppers).

Since changes in wine color have been reported and discussed in many previous studies, we present here only the results after 24 months of storage. The light absorbance at 420 nm (A420 nm) is conventionally used to measure the color characteristics of white wines, including the browning index. The warm aged samples had higher A420 nm values than the cool ones and both of these groups formed clusters in the CIELAB coordinates (Figure 6). These outcomes correlate with our recent research where the application of UV-Vis spectroscopy combined with sensory analysis was proposed as a potential approach for studying temperature-related influence on wine during transportation [23]. Comparing the impact of different wine bottling variants on wine color, the Riesling wines were quite similar visually. However, color analysis revealed a tendency for the *medium SO_2_* and *low SO_2_* samples to have somewhat higher values, at A420 nm and on the yellow color + b * axis in the CIELAB coordinates. In other studies of white wines, for instance Cortese wines, the samples without SO_2_ addition at bottling were more colored after only three months of storage than the ones with added SO_2_ [40].

### 3.3. C_13_-Norisoprenoids

**Figure 7:**
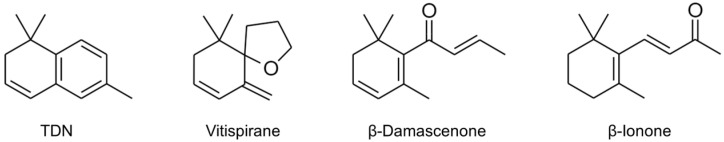
C_13_-norisoprenoids which can be found in Riesling wines.

The evolution of C_13_-norisoprenoids in Riesling wines is described in the literature in a fragmentary way only. The presence and development of the four following compounds in the studied wine (Figure 7) are discussed in the current Subsection.

**Figure 8:**
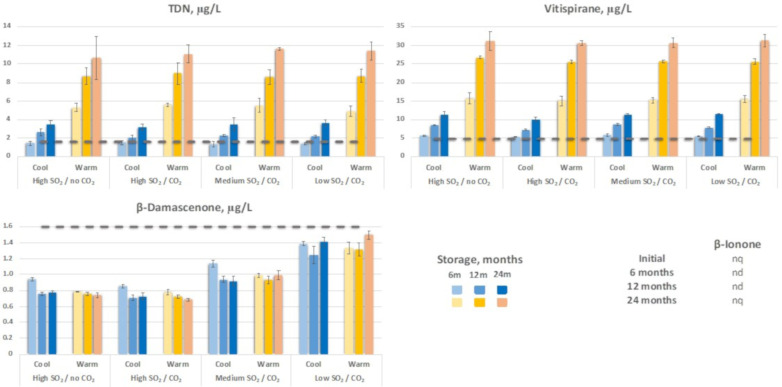
Concentration of C_13_-norisoprenoids in the wines after 6, 12 and 24 months of storage (mean of three samples with *Diam 5*, *Diam 30* and *Diam 30 origin* stoppers ± standard deviation; nd—nondetectable, nq—nonquantifiable). Dotted lines correspond to the initial levels of C_13_-norisoprenoids after bottling.

TDN is one of the most important substances among C_13_-norisoprenoids associated with the aging of Riesling wine and is characterized by a kerosene/diesel aroma. It has been demonstrated that a longer aging time and higher storage temperature favor the formation of TDN in wine [41,42]. In addition, it has been shown that nonpolar TDN molecules, in contrast with polar wine components, can be intensively absorbed by synthetic bottle closures and, to a lesser extent, by cork closures (“scalping effect”) [43,44,45,46]. The scalping properties of agglomerated closures towards TDN are usually between corks and plastic stoppers and depend on their composition [43,46]. In this study, based on the relatively small standard deviations, we assume that TDN was absorbed quite similarly by all three types of agglomerated *Diam* stoppers, despite some differences in their composition (Figure 8). This particularity of “scalping” a certain amount of TDN by bottle closures can be used as a tool to manage the TDN content in aged Riesling wines to avoid a high intensity of the kerosene/diesel aroma.

At the same time, it was not clear from the literature whether oxidation processes affect the TDN content in wine. One of the studies suggested that a certain amount of oxygen promotes the formation of TDN in port wines, while higher oxygen levels can intensify its degradation [47]. In this study, it was observed that a reduction of the oxygen content in the headspace (achieved by flushing with CO_2_), higher Free SO_2_ level in wine or higher OIR/OTR of *Diam 5* stoppers did not affect the TDN content during storage (Figure 8). Therefore, we assume that the formation or degradation of TDN in Riesling wines is not strongly related to the oxidation reactions, at least under the given bottling conditions. This assumption is also supported by the other study, in which the addition of antioxidant ascorbic acid to Riesling wine did not significantly influence the TDN level after 36 months of wine aging [44]. Storage temperature in the current study was the factor with the greatest impact on the TDN content. The temperature difference of 10 °C accelerated the TDN formation reactions by two to three times, which can also have a sensorial impact on the perception of the kerosene/diesel aroma in Riesling wine. Thus, after two years of storage under room (warm) conditions, the TDN concentration reached its recognition threshold of 10–12 µg/L [48].

The accumulation of vitispirane in Riesling wine had a similar to the TDN tendency. These compounds are biogenetically related, and the difference was related to their amounts: the initial and subsequent concentrations of vitispirane were about three times higher compared to that of TDN. Despite the vitispirane content reaching up to 34 µg/L, its sensorial impact is expected to be limited, since its detection sensory threshold was reported at approximately 100 µg/L [49]. In general, vitispirane is characterized by “camphor” and “eucalyptus” aromas. The positive correlations of heat during the wine storage with vitispirane and TDN contents were also reported before [50].

**Scheme 1:**
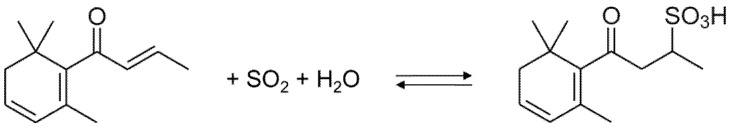
Reaction of β-damascenone with sulfur dioxide in wine (based on [51]).

In contrast to TDN and vitispirane, the content of β-damascenone did not depend much on the storage temperature, but was affected by bottling conditions. The wines with a *low SO_2_* level had about 50–100% more β-damascenone than the *high SO_2_* samples. A negative correlation was also found between β-damascenone and SO_2_ levels in the PCA analysis (Supplementary Figure S2). These outcomes are related to the ability of β-damascenone to react with sulfur dioxide, yielding mostly an odorless product by Michael-type addition, the derivative of sulfonic acid (Scheme 1) [51]. This interaction with sulfur dioxide is probably one of the most important transformations of β-damascenone in wine, since the higher oxygen content in the headspace did not lead to further losses of this compound. The typical odor of β-damascenone is often associated with “stewed apple” or “pear” aromas. This molecule is a very potent odorant and its odor threshold in a model wine was determined at 50 ng/L. However, in real wines the sensory threshold of β-damascenone can be 100-fold higher due to the impact of other matrix components [52]. The concentration of other important C_13_-norisoprenoid, β-ionone, was below the level of quantification already in the initial samples of Riesling wine.

### 3.4. Monoterpenes and Monoterpenoids

Monoterpenes and monoterpenoids are a wide group of C_10_-terpene compounds known for their contribution to floral and fruity aromas, particularly in young white wines. During the wine aging process, these compounds can be rearranged into other molecules with more complex scents. As in the case of C_13_-norisoprenoids, the mechanisms of their conversions in wine are not fully understood. In addition, it is not clear how oxidation reactions contribute to these transformations.

**Figure 9:**
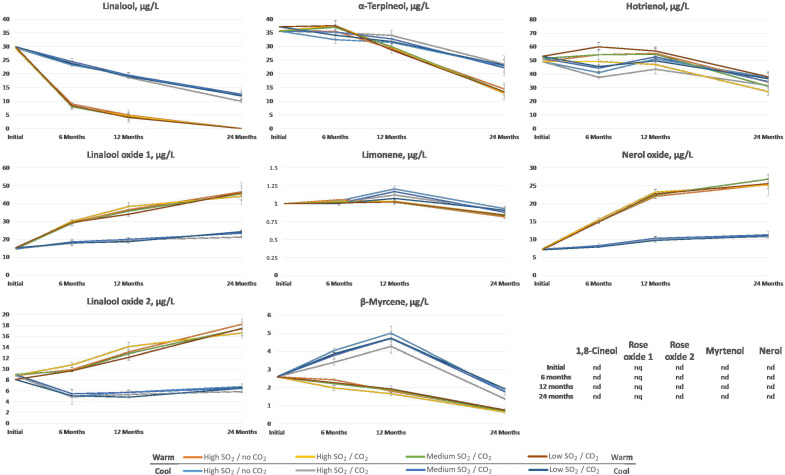
Concentration of monoterpenes and monoterpenoids in the wines after 6, 12 and 24 months of storage (mean of three samples with *Diam 5*, *Diam 30* and *Diam 30 origin* stoppers ± standard deviation).

**Scheme 2:**
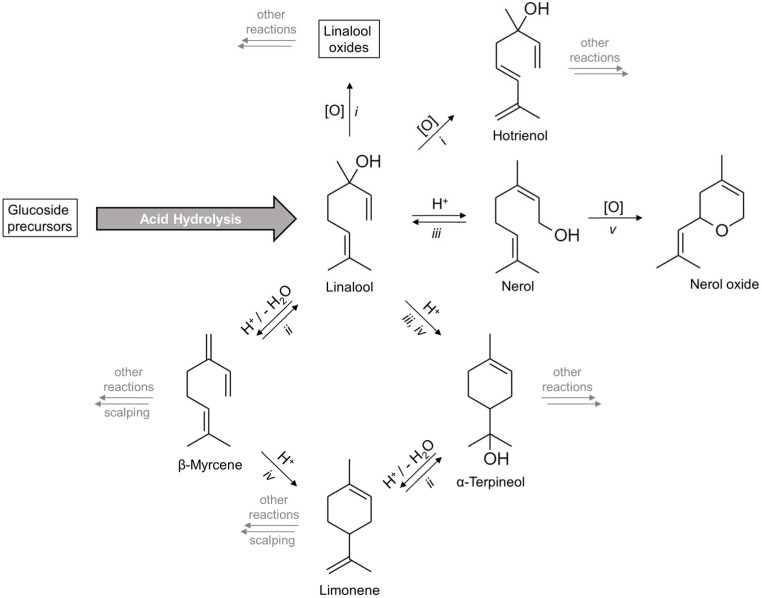
Proposed transformations of linalool in wine during the aging process (based on the previous publications [12,19] and our observations): (i) oxidation; (ii) acid-mediated dehydration; (iii) acid-mediated isomerization; (iv) acid-mediated cyclization; (v) oxidative cyclization.

Linalool is one of the most important monoterpenoids in *Vitis vinifera* grapes and corresponding young wines. Its initial concentration of about 30 µg/L in the studied Riesling wine was slowly decreasing under cool storage conditions and decreased more intensively at the higher temperature, eventually reaching the non-detectable values after 24 months (Figure 9). Linalool content is usually prevented from a rapid fall by the acid hydrolysis of its bound form (glucoside precursor). Cleavage of the O-Glc bond during hydrolysis yields extra amounts of free linalool (Scheme 2). One of the important reactions of linalool in wine is its oxidation with the formation of a series of linalool oxides: *cis*-and *trans*-forms of furano-and pyrano-isomers [19]. In the current experiment, the stereoisomers of furano-linalool oxides were analyzed. A relatively fast accumulation of these compounds at a higher temperature was found, especially for linalool oxide 1. The content of the latter also increased slowly at the lower temperature, while the concentration of linalool oxide 2 somewhat declined, which was probably due to further chemical transformations of these compounds. A similar tendency was observed in the *Vinhos Verdes* wines after 20 months of aging: furano-linalool oxides sharply increased in its level while pyrano-linalool oxides did not show a significant evolution [53]. The other proposed oxidative reactions of linalool include the formation of nerol oxide and hotrienol. The latter, apparently, also underwent subsequent conversions since its content began to decrease after 12 months. It is noteworthy that the reduction of O_2_ content in the bottle headspace did not affect the described oxidative transformations. This is probably because the amount of dissolved oxygen in all variants was sufficient for the oxidation reactions to proceed. At the same time, previous research with spiked linalool to Semillon wine showed that nitrogen in the headspace resulted in somewhat less degradation of this compound after two years [43]. Higher SO_2_ level in the wine also had a limited impact on the transformations of monoterpenes and monoterpenoids in the actual study. It did not preserve the content of linalool (no evident correlation was found between linalool and SO_2_ levels in the PCA analysis, Supplementary Figure S2) but tended to reduce the formation of hotrienol. In turn, the wine storage temperature was the most influential factor for the various transformations of monoterpenes and monoterpenoids.

Non-oxidative transformations of linalool can yield less odor-potent α-terpineol and then limonene [12,19]. We assume that the latter may also be produced from linalool via acid-mediated dehydration, followed by structural rearrangement (isomerization) of the formed β-myrcene (Scheme 2). Reverse formation of β-myrcene from limonene is a less likely reaction since it proceeds through ring-opening of stable cyclohexene moiety and requires the cleavage of the C-C bond. At cool storage conditions, the increase of β-myrcene content was observed during the first 12 months, followed by a fall to a level below the initial concentration. Apart from chemical reactions, the limited accumulation of β-myrcene and limonene can also be related to the scalping effect. Both of these terpenes are rather non-polar molecules due to the absence of polar hydroxyl groups (–OH). Therefore, various bottle closures may absorb these compounds, similarly to TDN [46]. The hydroxyl-containing polar monoterpenoids are much less susceptible to the scalping process, as was shown on the examples of linalool or nerol [43,45].

In addition, there were several monoterpenoids, which were not detected in the given Riesling wine: e.g., nerol, myrtenol and rose oxide. The latter is known to contribute to the aroma profiles of Gewurztraminer, Muscat grapes with rose and litchi notes. Rose oxide was also found in Riesling grapes; therefore it is worth monitoring and studying the development of this compound in grapes, then during enological processes and finally in wines, because of its high aroma potential.

### 3.5. Secondary Wine Aromas

**Figure 10:**
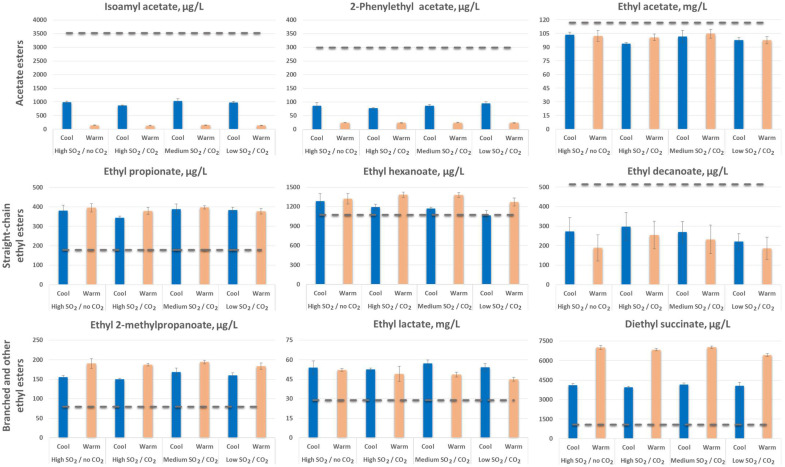
Concentration of selected esters in the wines after 24 months of storage (mean of three samples with *Diam 5*, *Diam 30* and *Diam 30 origin* stoppers ± standard deviation). Dotted lines correspond to the initial levels of esters after bottling (more details in Supplementary Table S2).

**Scheme 3:**
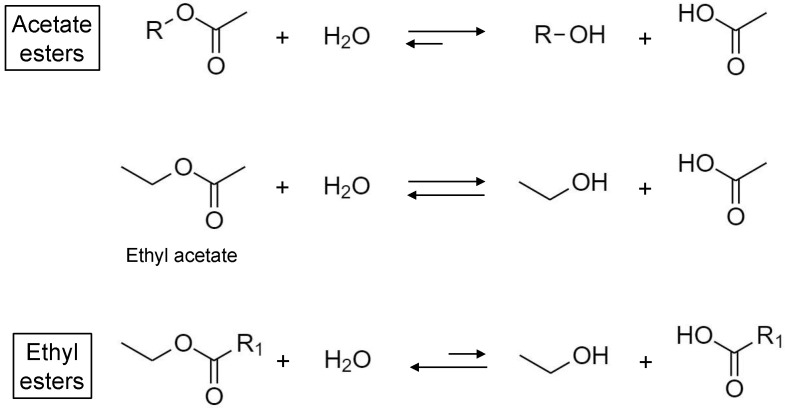
Hydrolysis and formation of esters in wine.

Hydrolysis of esters in wine is well described in the scientific literature, including the impact of different factors on this process, e.g., storage temperature or glass bottle hue [8,9,10,17]. Therefore, our study demonstrates here only the 24 month results of the secondary wine aromas development. In accordance with the previous studies, the concentration of acetate esters in the Riesling wine samples decreased considerably faster at a higher storage temperature (Figure 10). The greatest rate of hydrolysis was observed for hexyl acetate, the content of which reached non-quantifiable levels under warm storage conditions (Supplementary Table S2). Concentration of acetate esters in the cool stored samples also fell significantly, by about 3–4 times. The differences in bottling conditions (SO_2_ content in the wine and CO_2_ in the headspace) did not considerably affect the concentration of acetate esters, as well as ethyl esters. Considering ethyl acetate, its content descended only minorly and remained relatively stable for all the variants around 100 mg/L. These outcomes are explained by the high concentration of ethanol in wine, which maintains the ester’s formation/hydrolysis equilibrium and retains the high level of ethyl acetate (Scheme 3). In general, the formation of ethyl esters in wine is promoted by the excess of ethanol. However, the content of some ethyl esters decreased after 24 months, which may be due to other factors, such as the scalping effect. For example, ethyl decanoate with a long non-polar alkyl group was reported to be absorbed by cork and polymeric materials, especially by synthetic stoppers [43].

### 3.6. Low Molecular Weight Sulfur Compounds (LMWSCs)

**Figure 11:**
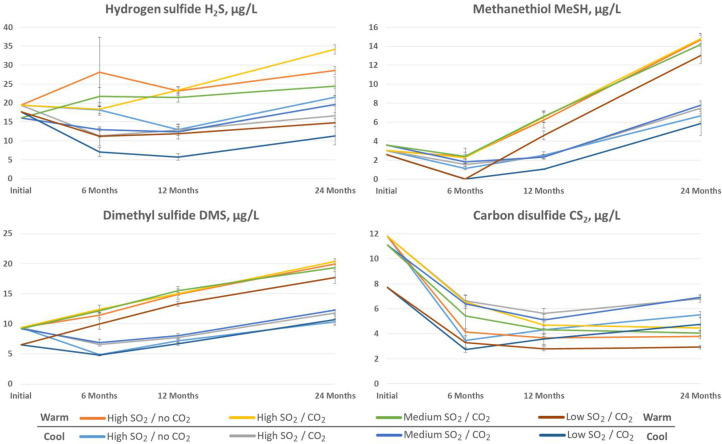
Concentration of LMWSCs in the wines after 6, 12 and 24 months of storage (mean of three samples with *Diam 5*, *Diam 30* and *Diam 30 origin* samples ± standard deviation).

Possible formation of unpleasant “reductive” aromas in wine is another important aspect of the aging process. LMWSCs are usually responsible for this wine defect and are characterized by off-odors, such as rotten egg, garlic, cabbage etc. [13]. Four LMWSCs presented in Figure 11 were found in the studied Riesling wine initially and in the subsequent stages of storage. The opposite chemical concept of “reduction” is “oxidation”. Therefore, it was of great interest to monitor the development of LMWSCs in the wine variants with lower and higher oxygen contents, as well as with different levels of antioxidant SO_2_.

**Scheme 4:**
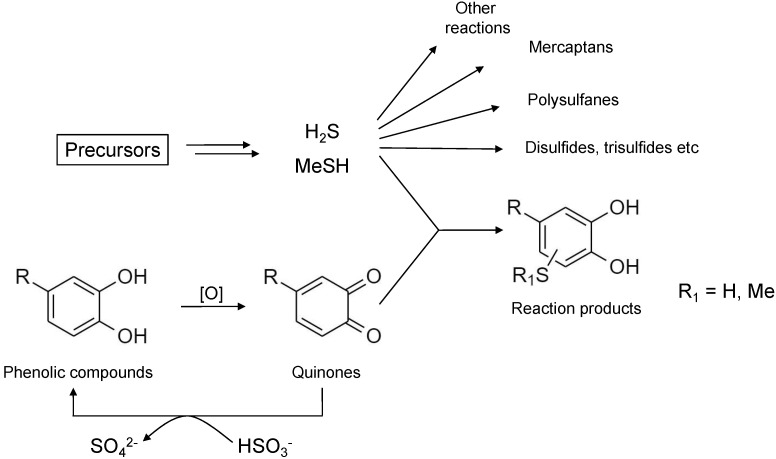
Formation of LMWSCs (H_2_S and MeSH) and their reactions with wine components.

Among LMWSCs, H_2_S and MeSH are most often referred to as reductive notes in wine. The formation mechanisms of these compounds in wine have not yet been clearly defined. The amino acids, cysteine and methionine, are considered among the putative precursors of H_2_S and MeSH [54]. Reactions related to the reduction of sulfates and SO_2_ have also been suggested as a source of H_2_S in wine during the bottle aging [55]. In general, two opposite processes affect the LMWSCs content in wine: the formation of LMWSCs and their reactions with various wine components. H_2_S and MeSH are strong nucleophiles that readily react with electrophiles in situ generated by the oxidative processes in wine, e.g., quinones [56] and other carbonyl compounds (Scheme 4). In addition, they can be converted into disulfides and trisulfides and participate in the formation of mercaptans (e.g., furfuryl mercaptan, benzyl mercaptan) [13], complex polysulfanes and polythionates [57].

In the present study, lower levels of H_2_S and MeSH were observed in the variants with *low SO_2_* at all stages of wine storage. In the case of MeSH, its concentration decreased during the first six months, which correlates with the consumption of the dissolved oxygen in wine in this period. Then the reactions of MeSH formation began to prevail, and this compound demonstrated a rapid accumulation, especially under warm storage conditions. A similar trend was observed in Shiraz wine, when MeSH accumulated much faster, starting from the sixth month of storage [58]. The differences in SO_2_ levels in the studied Riesling wine did not show large variations in the MeSH level after 24 months (no correlation was found between MeSH and SO_2_ levels in the PCA analysis, Supplementary Figure S2). At the same time, a more heterogeneous picture was demonstrated by H_2_S, which can be due to its reactivity with a wider set of other wine components. Thus, the greatest variation between the samples was after six months of storage. After 12 months, the H_2_S levels in the *high SO_2_* and *medium SO_2_* variants were very close, while the *low SO_2_* variants contained about half as much H_2_S at each temperature setting. Analysis at 24 months again revealed a certain variation: the highest H_2_S level among the warm stored samples corresponded to the *High SO_2_/CO_2_* variant; the cool aged wines had somewhat higher level of hydrogen sulfide in the *High SO_2_/noCO_2_* samples. Together with previous studies [18,21], the current results of H_2_S deviation demonstrated that the management of hydrogen sulfide in wine has no straightforward solutions and depends on many factors, including Cu ions and glutathione content [18]. The aroma threshold values for LMWSCs deviate quite significantly in the literature, e.g., the range for H_2_S is 1.1–80 µg/L [7,59]. Despite the elevated content of the analyzed LMWSCs, no considerable reductive notes were found in the tasted samples in the sensory tests, which will be discussed in detail in the next Subsection.

The analysis of DMS showed its constant accumulation in the warm stored wine samples. Under cool conditions, the wines first lost some amount of DMS, and its level then recovered and, after 24 months, slightly exceeded the original content. As in the research with Shiraz wines [58], the higher exposure to oxygen did not considerably affect the DMS content in the studied wines. Regarding the influence of SO_2_ level in wine, all variants demonstrated a similar rate of DMS accumulation at each temperature setting. DMS can also contribute to the reductive impression in wine with popcorn and molasses aromas, but this was not the case in the tasted samples. In turn, CS_2_ is a much less prominent scent contributor compared to the LMWSCs described above. Moreover, its content declined already after six months and continued to remain below the original level. In contrast to other LMWSCs, the CS_2_ concentration after 24 months was somewhat higher in the cool stored samples than in the warm aged counterparts. Reactions of CS_2_ in wine are also to be studied in the future.

### 3.7. Sensory Analysis

Sensory analysis was one of the key steps of this study. The discussed above results of the physicochemical analyses were focused mainly on changes in the varietal and secondary wine aromas’ composition, which were the main interest of this research. Apart from the determination of acetaldehyde, there was no intention to analyze the whole spectra of compounds related to the oxidized wine aroma, such as sotolon, furfural, phenylacetaldehyde, benzaldehyde, methional, isobutyraldehyde and other carbonyl compounds [13,60,61]. Therefore, sensory analysis was an important instrument for monitoring the overall changes associated with the wine aging and oxidation processes.

The sensory analysis of the current work comprised two parts. On the first step, the goal was to check whether the Riesling wines sealed with various stoppers, *Diam 5*, *Diam 30* and *Diam 30 origin*, produced any differences in the aroma bouquet. Ranking tests were utilized for this purpose and were offered to arrange the wine samples according to their oxidation state. The results of these tests are presented in the Supplementary Figure S3 and it can be concluded that no differences in the aroma composition were observed between the wines with different stoppers, even after two years. These results are not surprising since no important differences between these wines were detected also in the physicochemical analyses and all of the tested Diam stoppers are designed for a long wine storage, at least 5 and 30 years, respectively. Statistical differences, which were found in two tests after six months, were of the lowest significance and could be influenced by other factors than the stoppers’ properties. Both of these tests included the most protected wine variants with *high SO_2_* level stored at cool conditions. Therefore, the found deviations can be related to a slight reductiveness generated by SO_2_ and a pungent smell of the latter. In the subsequent tests, no statistical differences were found for these variants after 12 and 24 months.

**Figure 12:**
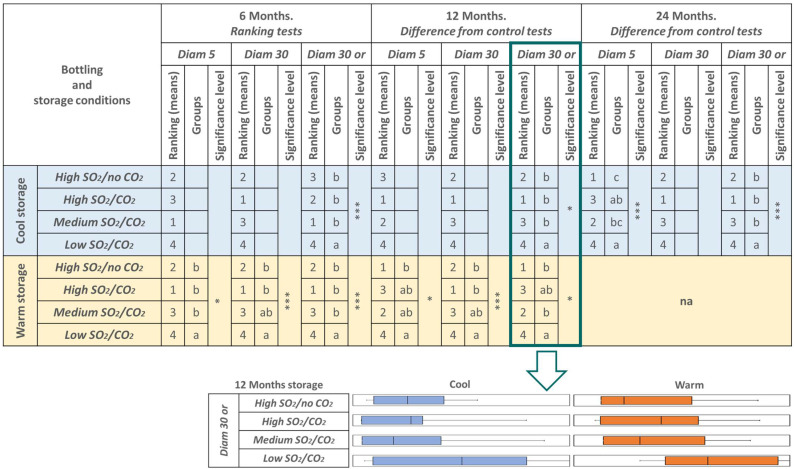
Results of sensory analysis of the wines after 6, 12 and 24 months of storage: comparison of bottling and storage conditions effects. An example of the highlighted *difference from control test* (median is indicated by the vertical lines; the left and right sides of the box represent the 1st and 3rd quartiles, respectively; the whiskers represent the minimum and maximum values). Asterisks represent significant differences within each sensory test: * *p* < 0.05; *** *p* < 0.001; empty cell—no significant differences.

The second pool of sensory tests was focused on the possible differences in wine aromas related to the variation of bottling conditions (Figure 12). As in the previous tests, the panelists were asked to arrange the wine samples according to their oxidation state. It is clear from the outcomes that the wines with a *low SO_2_* level stored at warm conditions were perceived as more oxidized and nutty already after only six months. These results correlate with the study of white Cortese wines stored at 20 °C [40]. The oxidized aromas in our Riesling samples were similar to the ones generated by Strecker aldehydes and sotolon. The analogous tendency was observed also for the cool aged wines when the *low SO_2_* variants always received the fourth rank, but mostly without statistical significance after 6 and 12 months. An example of *difference from control tests* (12 months of storage, *Diam 30 origin* stoppers) illustrates the evaluation of the cool and warm stored wines using box plots (Figure 12). The median of the cool *low SO_2_* variant was about twice as high as for other samples. At the same time, the difference between the first and third quartiles was quite large. This means that, in general, the tasters as a group identified the more oxidized character of the *low SO_2_* wine, but some of the panelists still had complications in distinguishing it from the other samples. With regard to the warm stored wines, the panelists’ answers were more grouped when evaluating the *low SO_2_* sample.

In the 24-months *difference from control tests*, the *low SO_2_* cool wines were discriminated with a high level of significance in two of the three ranking tests. In turn, no sensory analyses were performed for the warm aged wines after 24 months, since the relatively intense oxidation was perceived already after 6 and 12 months. Regarding the *high SO_2_* and *medium SO_2_* wines stored at cool temperature, none of these variants were perceived as oxidized, even after 24 months. However, these samples were different, and it was challenging to reach a consensus in terms of their freshness, since the fruitiness in the wines with higher SO_2_ could be covered by certain reductiveness related to sulfur dioxide effects. Therefore, no clear ranking differences were found between the *high SO_2_* and *medium SO_2_* samples stored at cool conditions, at least after 6 and 12 months. In the case of warm aged wines, the *medium SO_2_* variants tended to be evaluated as developed, occupying third place in the six months *ranking tests*, even though no statistical significance was found between the *medium SO_2_* and *high SO_2_* samples.

## 4. Conclusions

This study was focused on the development of Riesling wine during bottle aging, depending on a series of bottling conditions (*Free SO_2_* level in wine: *low*—*13 mg/L*, *medium*—*24 mg/L*, *high*—*36 mg/L*; CO_2_ treatment of the headspace), storage conditions (warm room~25 °C vs. cool cellar ~15 °C) and type of stoppers (*Diam 30*, *Diam 30 origin* and *Diam 5* with the OIR range 0.8–1.3 mg and OTR range 0.3–0.4 mg/year). It was shown that storage temperature was the main factor affecting transformations of varietal and secondary aromas in the wine. At the same time, reduction of the oxygen content in the bottles’ headspace, lower OIR/OTR of the stoppers and higher Free SO_2_ levels in the wine did not have a considerable impact on the content of majority of monoterpenoids (linalool, α-terpineol etc.), C_13_-norisoprenoids (TDN, vitispirane) and esters after 6–24 months of storage. These facts reveal new aspects about the impact of oxidation reactions on the stability and formation of these compounds during the wine aging process. Nevertheless, the wine samples with low Free SO_2_ were perceived in the sensory tests as more oxidized already after six months. This demonstrates the sensorial predominance of oxidized aroma compounds in the wine bouquet, even if varietal and secondary aroma substances remain relatively stable. Considering LMWSCs, their development was also influenced mostly by the storage temperature. The reduction of the SO_2_ level in the wine had the greatest impact on the H_2_S content by lowering it. In addition, the H_2_S concentration seemed to also depend on other factors, which were not in the scope of this study. Finally, it was shown that all three types of micro-agglomerated Diam stoppers were able to preserve the Riesling wine during two years of storage in a very similar way. In general, no noticeable sensory differences were found between the variants with different stoppers, and the results of the physicochemical analyses were very close.

## Supplementary Materials

The following are available online, Supplementary Table S1. Basic wine parameters in the initial wine and in the wine samples after 6, 12 and 24 months of storage; Supplementary Table S2. Concentration of the secondary wine aroma in the initial wine and after 24 months of storage; Supplementary Figure S1A. Extracted Ion Chromatogram: TDN and naphthalene-*d_8_* (internal standard); Supplementary Figure S1B. Extracted Ion Chromatogram: linalool and linalool-*d_3_* (internal standard); Supplementary Figure S2. Results of PCA analysis of the wines after 24 months of storage: SO_2_ content, terpenes and C_13_-norisoprenoids (varietal aromas), LMWSCs; Supplementary Figure S3. Results of sensory analysis of the wines after 6, 12 and 24 months of storage: comparison of *Diam 5*, *Diam 30* and *Diam 30 origin* bottle stoppers effect in *ranking tests*.

## Sample Availability

Limited availability of the wine samples from the authors.
