# Supplementary material for: Wine Storage at Cellar vs. Room Conditions: Changes in the Aroma Composition of Riesling Wine
Source: Molecules. 2021 Oct 16;26(20):6256. doi: 10.3390/molecules26206256 (PMC8540336; doi:10.3390/molecules26206256)
Supplement: Supplementary file 1 [file molecules-26-06256-s001.zip › molecules-1281439-supplementary.pdf]

Supplementary Table S1. Basic wine parameters in the initial wine and in the wine samples after 6, 12 and 24 months of storage

Parameter[1]	Units	Analysis after bottling	Analysis after storage time[2,3,4]					
			6 months		12 months		24 months	
			Cool	Warm	Cool	Warm	Cool	Warm
Density	20/20	0.9956 ± 0.0002	0.9957 ± $3*10^{-16}$		0.9955 ± 0.0004		0.9956 ±0.0003	
Alcohol	g/L	98.4 ± 1.1	97.9 ± 0.9		98.7 ± 1.0		97.8 ± 0.9	
Glycerol	g/L	5.6 ± 0.1	5.6 ± 0.1		5.7 ± 0.1		5.6 ± 0.1	
Extract	g/L	28.7 ± 0.1	27.8 ± 0.1	29.3 ± 0.2	28.3 ± 0.2	30.1 ± 0.3	29.5 ± 0.3	31.2 ± 0.4
Sugar-free Extract	g/L	21.6 ± 0.2	20.7 ± 0.2	22.3 ± 0.2	21.1 ± 0.2	22.9 ± 0.2	22.3 ± 0.3	24.1 ± 0.4
Fermentable Sugars	g/L	7.1 ± 0.1	7.1 ± 0.1		7.2 ± 0.1		7.1 ± 0.1	
Glucose	g/L	3.3 ± 0.1	3.2 ± 0.1		3.4 ± 0.1		3.5 ± 0.1	
Fructose	g/L	3.8 ± 0.1	3.8 ± 0.1		3.8 ± 0.1		3.7 ± 0.1	
pH		3.5 ± 0	3.5 ± 0		3.4 ± 0		3.4 ± 0.1	
Total acidity	g/L	7.2 ± 0.1	6.9 ± 0.1	7.3 ± 0.1	6.9 ± 0.1	7.4 ± 0.1	6.9 ± 0.1	7.3 ± 0.1
Tartaric acid	g/L	2.6 ± 0.1	2.2 ± 0.1	2.7 ± 0.1	2.1 ± 0.0	2.7 ± 0.0	1.9 ± 0.1	2.5 ± 0.1
Malic acid	g/L	4.1 ± 0	4.0 ± 0.1		4.1 ± 0.1		4.1 ± 0.1	
Lactic Acid	g/L	0.4 ± 0.1	0.4 ± 0.1		0.4 ± 0.1		0.4 ± 0.1	
Volatile acidity	g/L	0.6 ± 0	0.6 ± 0		0.6 ± 0		0.6 ± 0.1	
Acetaldehyde	mg/L	na	24.5 ± 0.5	25.7 ± 1.6	23.7 ± 0.6	24.3 ± 0.7	24.3 ± 0.6	24.9 ± 0.5

[1] All the parameters were measured by FTIR analysis, except the acetaldehyde content, which was measured by the enzymatic method

[2] Values on the blue background: mean of all wine samples' variants stored at Cool conditions ± standard deviation

[3] Values on the yellow background: mean of all wine samples' variants stored at Warm conditions ± standard deviation

[4] Values on the white background: mean of all wine samples' variants stored at Cool and Warm conditions ± standard deviation

Supplementary Table S2. Concentration of the secondary wine aroma in the initial wine and after 24 months of storage

Group aromas	Aroma compound	Initial wines[1]	Wines after 24 months of storage[2]							
			High SO_2/no CO_2		*High SO_2/CO_2*		*Medium SO_2/CO_2*		*Low SO_2/CO_2*	
			Cool	Warm	Cool	Warm	Cool	Warm	Cool	Warm
Acetate esters	Isoamyl acetate, µg/L	3528 ± 319	983 ± 42	142 ± 12	873 ± 13	131 ± 9	1037 ± 80	146 ± 15	979 ± 39	134 ± 10
	2-Methylbutyl acetate, µg/L	166 ± 17	49 ± 3	21 ± 1	44 ± 0	20 ± 1	51 ± 5	20 ± 1	50 ± 2	20 ± 0
	Hexyl acetate, µg/L	221 ± 25	35 ± 6	nq	32 ± 3	nq	38 ± 7	nq	37 ± 5	nq
	2-Phenylethyl acetate, µg/L	299 ± 9	87 ± 11	25 ± 0	78 ± 2	24 ± 1	86 ± 6	25 ± 1	95 ± 6	24 ± 1
	Ethyl acetate, mg/L	117 ± 5	104 ± 3	102 ± 6	94 ± 1	101 ± 4	102 ± 7	105 ± 5	98 ± 3	98 ± 4
Ethyl esters	Ethyl propionate, µg/L	179 ± 17	381 ± 28	395 ± 22	343 ± 10	380 ± 18	388 ± 27	397 ± 9	384 ± 15	378 ± 14
	Ethyl butanoate, µg/L	695 ± 68	626 ± 16	598 ± 29	566 ± 19	578 ± 16	655 ± 41	599 ± 13	627 ± 24	565 ± 22
	Ethyl hexanoate, µg/L	1076 ± 163	1284 ± 120	1322 ± 79	1195 ± 43	1384 ± 40	1169 ± 22	1380 ± 36	1066 ± 74	1273 ± 62
	Ethyl octanoate, µg/L	1468 ± 160	1084 ± 224	1084 ± 111	1054 ± 114	1271 ± 5	910 ± 22	1207 ± 44	815 ± 152	1054 ± 81
	Ethyl decanoate, µg/L	515 ± 50	272 ± 71	188 ± 68	298 ± 72	255 ± 70	270 ± 53	232 ± 74	221 ± 41	186 ± 57
	Ethyl 2-methylpropanoate, µg/L	80 ± 11	156 ± 4	191 ± 13	150 ± 3	187 ± 4	169 ± 10	194 ± 4	160 ± 6	184 ± 8
	Ethyl 2-methylbutyrate, µg/L	na	25 ± 2	41 ± 2	24 ± 1	40 ± 1	28 ± 2	40 ± 1	26 ± 1	40 ± 1
	Ethyl 2-hydroxy-4-methylvalerate, µg/L	nq	132 ± 13	130 ± 2	125 ± 4	125 ± 7	139 ± 1	128 ± 5	144 ± 17	122 ± 2
	Ethyl phenylacetate, µg/L	nq	12 ± 1	13 ± 0	11 ± 0	12 ± 0	12 ± 1	13 ± 0	12 ± 1	13 ± 0
	Ethyl lactate, mg/L	29 ± 1	54 ± 5	52 ± 1	53 ± 1	49 ± 6	57 ± 3	49 ± 2	54 ± 3	45 ± 1
	Diethyl succinate, µg/L	1096 ± 18	4118 ± 130	6998 ± 153	3954 ± 75	6831 ± 83	4161 ± 114	7017 ± 92	4072 ± 264	6438 ± 119
Higher alcohols	*i*-Butanol, mg/L	33 ± 2	30 ± 3	32 ± 1	28 ± 1	30 ± 2	30 ± 2	30 ± 1	30 ± 0	30 ± 2
	Isoamyl alcohol, mg/L	162 ± 8	152 ± 11	162 ± 6	141 ± 8	161 ± 6	138 ± 6	161 ± 5	131 ± 9	152 ± 6
	2-Methyl-1-butanol, mg/L	30 ± 2	31 ± 1	31 ± 1	28 ± 1	30 ± 2	29 ± 2	30 ± 1	29 ± 1	30 ± 1
	Hexanol, µg/L	1152 ± 44	1672 ± 117	1567 ± 59	1512 ± 45	1456 ± 71	1664 ± 120	1534 ± 40	1713 ± 85	1497 ± 34
	2-Phenylethanol, mg/L	19 ± 0	24 ± 0	24 ± 1	22 ± 0	23 ± 1	23 ± 1	24 ± 0	24 ± 0	23 ± 0
Fatty acids	*i*-Valeric acid, µg/L	1717 ± 25	1237 ± 117	1102 ± 22	1338 ± 22	1053 ± 29	1378 ± 40	1104 ± 21	1390 ± 107	1085 ± 10
	Hexanoic acid, mg/L	12 ± 0	8 ± 1	8 ± 0	8 ± 0	7 ± 0	9 ± 0	7 ± 0	9 ± 1	7 ± 0
	Octanoic acid, mg/L	10 ± 0	8 ± 1	7 ± 0	7 ± 0	6 ± 0	8 ± 1	7 ± 1	8 ± 1	7 ± 1
	Decanoic acid, µg/L	2390 ± 270	1673 ± 439	1282 ± 81	1523 ± 156	1275 ± 136	1778 ± 343	1305 ± 109	1870 ± 330	1299 ± 118

[1] Mean of three Riesling wines after the adjustment of Free SO_2 level (*High SO_2*, *Medium SO_2*, *Low SO_2*) ± standard deviation
[2] Mean of three samples with *Diam 5*, *Diam 30* and *Diam 30 origin* stoppers ± standard deviation

Supplementary Figure S1A. Extracted Ion Chromatogram: TDN and naphthalene-d_8 (internal standard)

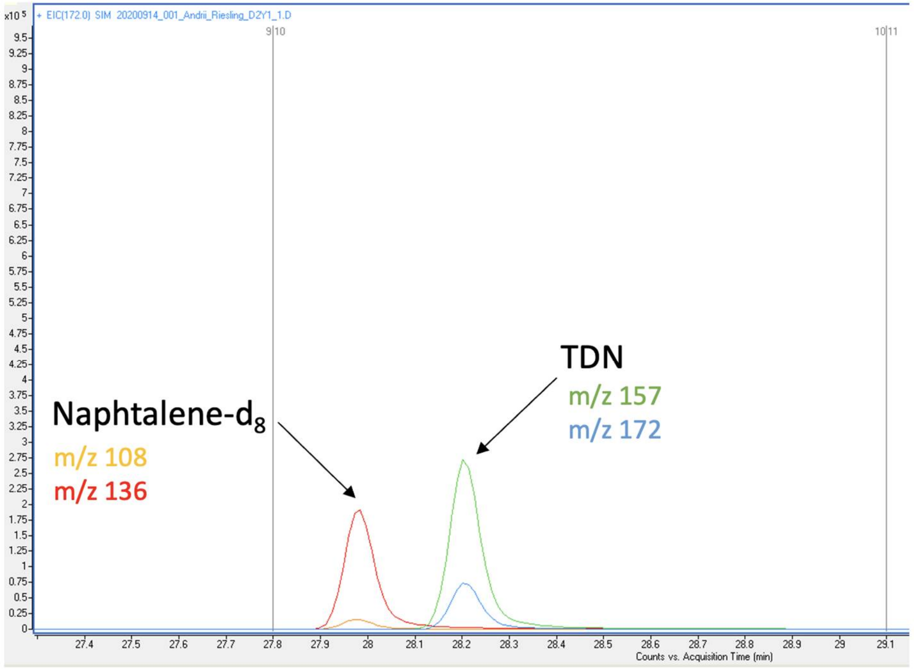

24 months: High SO_2/CO_2/Cool/Diam 5

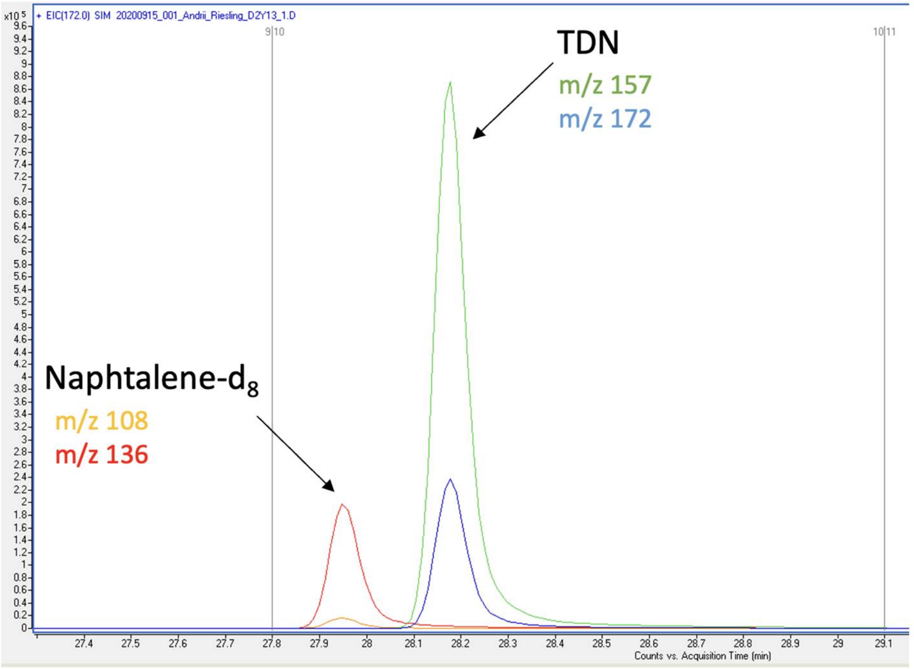

24 months: High SO_2/CO_2/Warm/Diam 5

Supplementary Figure S1B. Extracted Ion Chromatogram: linalool and linalool-d_3 (internal standard)

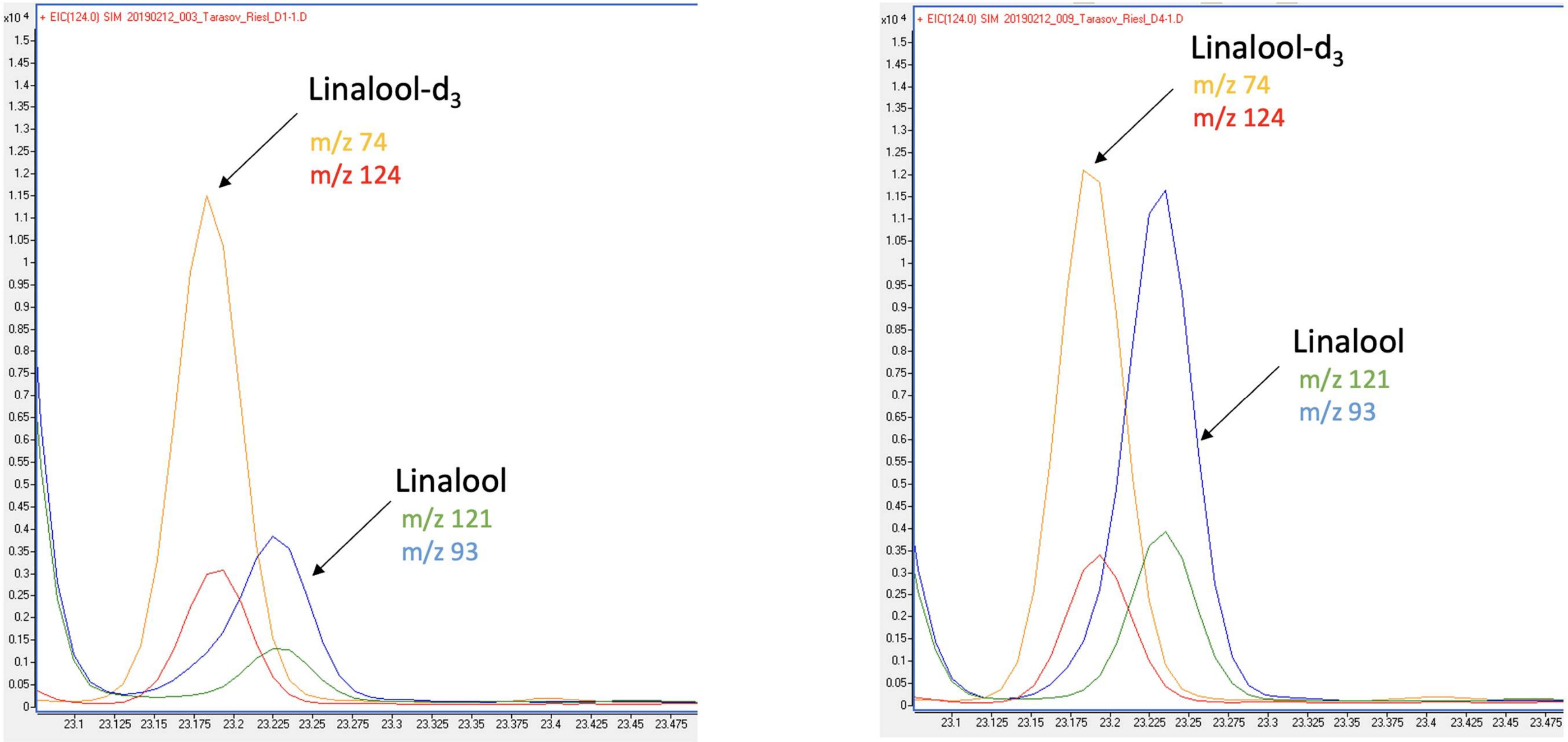

6 months: High SO_2/CO_2/Warm/Diam 5

6 months: High SO_2/CO_2/Cool/Diam 5

Supplementary Figure S2. Loadings plot for the PCA analysis of the wines after 24 months of storage: SO_2 content, terpenes and C_{13}-norisoprenoids (varietal aromas), LMWSCs

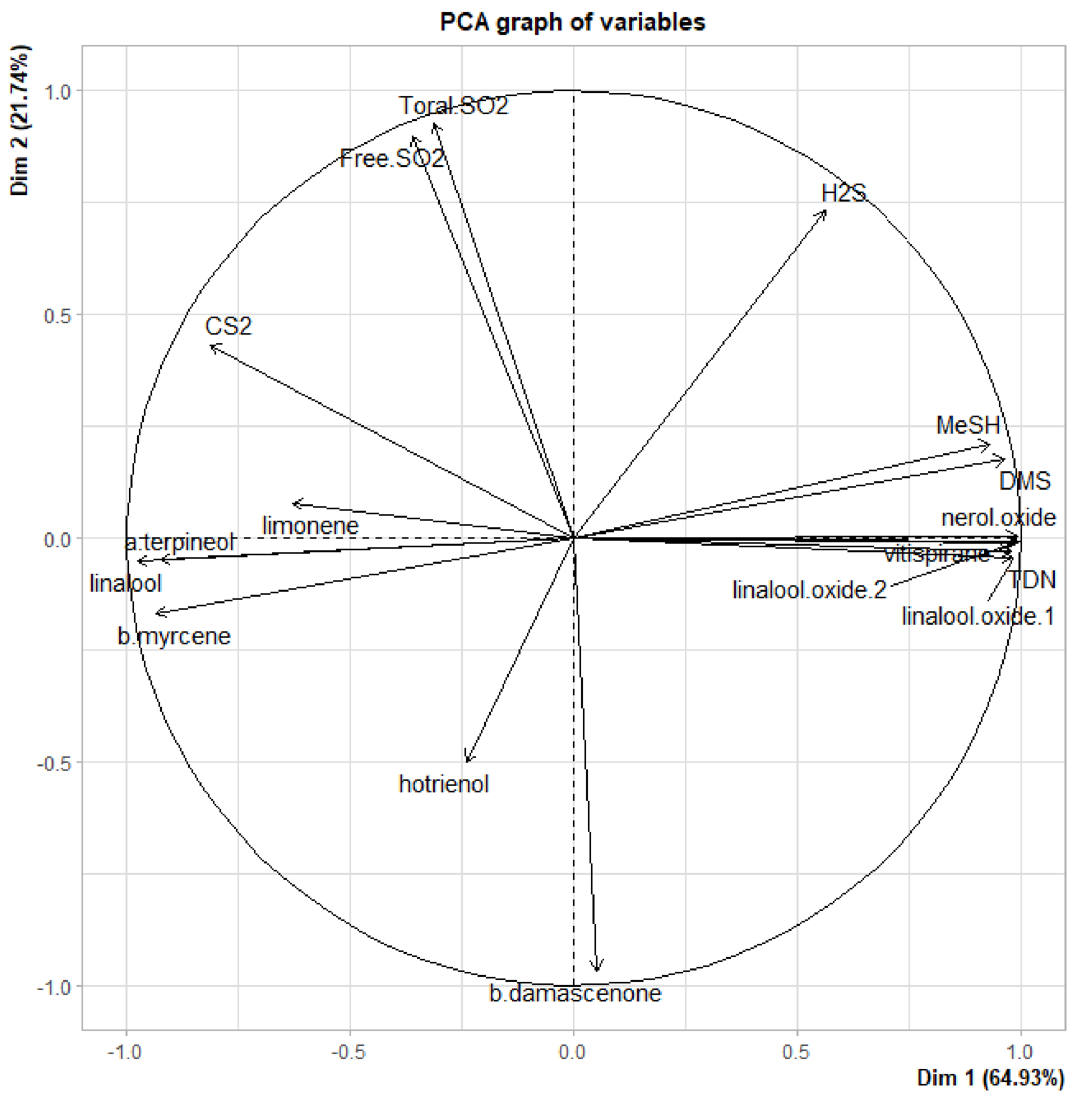

Supplementary Figure S3. Results of sensory analysis of the wines after 6, 12 and 24 months of storage: comparison of *Diam 5*, *Diam 30* and *Diam 30 origin* bottle stoppers effect in *ranking tests*

6 Months. *Ranking tests*

Bottling and storage conditions		High SO_2 / no CO_2 Ranking (means)	Groups	Significance level	High SO_2 / CO_2 Ranking (means)	Groups	Significance level	Medium SO_2 / CO_2 Ranking (means)	Groups	Significance level	Low SO_2 / CO_2 Ranking (means)	Groups	Significance level
Cool storage	*Diam 5*	2	a		1	b		3			2		
	Diam 30	2	a	*	3	a	*	1			1		
	Diam 30 or	1	b		2	b		2			1		
Warm storage	*Diam 5*	1			2			3			1		
	Diam 30	2			1			2			2		
	Diam 30 or	3			3			1			3		

12 Months. *Ranking tests*

Bottling and storage conditions		High SO_2 / no CO_2 Ranking (means)	Groups	Significance level	High SO_2 / CO_2 Ranking (means)	Groups	Significance level	Medium SO_2 / CO_2 Ranking (means)	Groups	Significance level	Low SO_2 / CO_2 Ranking (means)	Groups	Significance level
Cool storage	*Diam 5*	3			2			1			2		
	Diam 30	1			3			3			1		
	Diam 30 or	2			1			2			3		
Warm storage	*Diam 5*	1			1			2			1		
	Diam 30	3			3			1			2		
	Diam 30 or	2			2			3			3		

24 Months. *Ranking tests*

Bottling and storage conditions		High SO_2 / no CO_2 Ranking (means)	Groups	Significance level	High SO_2 / CO_2 Ranking (means)	Groups	Significance level	Medium SO_2 / CO_2 Ranking (means)	Groups	Significance level	Low SO_2 / CO_2 Ranking (means)	Groups	Significance level
Cool storage	*Diam 5*	1			3			2			2		
	Diam 30	2			1			3			1		
	Diam 30 or	3			2			1			2		
Warm storage	*Diam 5*	na											
	Diam 30												
	Diam 30 or												
